# Analysis of factors influencing hospitalization cost of patients with distal radius fractures: an empirical study based on public traditional Chinese medicine hospitals in two cities, China

**DOI:** 10.1186/s12913-024-10953-w

**Published:** 2024-05-09

**Authors:** Mengen Chen, Jingyu Yang, Haojia Hou, Baozhu Zheng, Shiji Xia, Yuhan Wang, Jing Yu, Guoping Wu, Henong Sun, Xuan Jia, Hao Ning, Hui Chang, Xiaoxi Zhang, Youshu Yuan, Zhiwei Wang

**Affiliations:** 1 School of Traditional Chinese Medicine, Beijing University of Chinese Medicine, Beijing, 102400 China; 2 School of Management, Beijing University of Chinese Medicine, Beijing, 102400 China; 3 School of Health Management, Gansu University of Chinese Medicine, Lanzhou, 730000 China; 4 School of Public Health, Lanzhou University, Lanzhou, 730000 China; 5 School of Public Health, Gansu University of Chinese Medicine, Lanzhou, 730000 China; 6 School of Stomatology, Capital Medical University, Beijing, 100050 China; 7 Guang’anmen Hospital, China Academy of Chinese Medicine Sciences, Beijing, 100053 China; 8 National Institute of Chinese Medicine Development and Strategy, Beijing, 102400 China

**Keywords:** DRFs, TCM^a^ hospitals, TCM^b^ advantageous diseases, Hospitalization cost

## Abstract

**Background:**

Distal radius fractures (DRFs) have become a public health problem for all countries, bringing a heavier economic burden of disease globally, with China’s disease economic burden being even more acute due to the trend of an aging population. This study aimed to explore the influencing factors of hospitalization cost of patients with DRFs in traditional Chinese medicine (TCM^a^) hospitals to provide a scientific basis for controlling hospitalization cost.

**Methods:**

With 1306 cases of DRFs patients hospitalized in 15 public TCM^a^ hospitals in two cities of Gansu Province in China from January 2017 to 2022 as the study object, the influencing factors of hospitalization cost were studied in depth gradually through univariate analysis, multiple linear regression, and path model.

**Results:**

Hospitalization cost of patients with DRFs is mainly affected by the length of stay, surgery and operation, hospital levels, payment methods of medical insurance, use of TCM^a^ preparations, complications and comorbidities, and clinical pathways. The length of stay is the most critical factor influencing the hospitalization cost, and the longer the length of stay, the higher the hospitalization cost.

**Conclusions:**

TCM^a^ hospitals should actively take advantage of TCM^b^ diagnostic modalities and therapeutic methods to ensure the efficacy of treatment and effectively reduce the length of stay at the same time, to lower hospitalization cost. It is also necessary to further deepen the reform of the medical insurance payment methods and strengthen the construction of the hierarchical diagnosis and treatment system, to make the patients receive reasonable reimbursement for medical expenses, thus effectively alleviating the economic burden of the disease in the patients with DRFs.

**Supplementary Information:**

The online version contains supplementary material available at 10.1186/s12913-024-10953-w.

## Introduction

DRFs are defined as a fracture within 3 cm from the articular surface of the distal radius [1], which is a relatively common type of fracture, most commonly seen in elderly women and children, whereas the occurrence of young adults is traumatic violence greater [2, 3]. A fracture of the distal radius may be described as a Colles, Smith, Barton, or Hutchinson fracture depending on the characteristics of the injury [2, 4]. In addition to these four commonly used fracture names, some specially named fractures have been deferred in clinical work, such as chauffeur’s fracture, die-punch fracture, and so on [5, 6].

Patients with DRFs account for approximately one-sixth of all fractures in the United States emergency departments, with an annual incidence of more than 640,000 cases [7], costing roughly $170 million in 2007 in Medicare reimbursement alone [8], and the incidence of DRFs in the United States is expected to continue to rise based on the evidence from existing studies [9, 10]. In addition to the U.S., available studies also show that the incidence of DRFs in countries and regions such as the United Kingdom, Finland, Sweden, and Norway will continue to increase over the coming period [11–14]. In China, DRFs account for about 20% of emergency fractures and 75% of forearm fractures, and the number of distal radius fracture patients may exceed 200 million by 2025 with the aging of China’s population [15–17]. DRFs have become a public health problem that places a heavy economic burden on people around the world, with fewer studies of DRFs in TCM^a^ hospitals being conducted to alleviate this problem.

TCM^a^ is the main component of Chinese medicine with a long history of development and is characterized by Chinese cultural connotations and local characteristics. In the process of its development, TCM^b^ has gradually formed characteristic therapies and methods for the treatment of some diseases, and these diseases are named ‘TCM^b^ advantageous diseases’. DRFs is one of the advantageous diseases in TCM^b^, treated as one of the key specialties in TCM^a^ hospitals. The treatment of DRFs in TCM^a^ hospitals can be broadly divided into two categories, one is the non-surgical treatment through reduction maneuvers and splinting, which is also the conservative treatment actively adopted by TCM^a^ hospitals, and the other is the surgical treatment through the fixation of the bone position using plate or stent to gradually achieve the healing of the injury [1, 18–21]. When the patient’s fracture condition is not serious, the TCM^a^ hospitals tend to promote the conservative treatment without secondary surgery of removing the plate, which generates less consumption of medical resources, supplementing with Chinese medicine to cooperate with the treatment can significantly improve the speed of recovery, reduce the length of stay, and ultimately reduce the hospitalization cost of the patient effectively.

The Chinese government is currently pushing forward the reform of medical insurance payment methods to improve the quality of medical services as well as to control medical costs, and the TCM^b^ advantageous diseases will be taken as the main target and direction of the preliminary reform in the exploration of the payment reform of TCM^a^ hospitals. The TCM^b^ advantageous diseases of DRFs is a common type of fracture in various countries with a high incidence of disease, analyzing and researching the factors affecting its hospitalization cost has great significance for health economics and public health, especially in the context of the Chinese government’s policy of comprehensively implementing the reform of the diagnosis related groups (DRG) or diagnosis-intervention packet (DIP) medical insurance payment methods with the TCM^b^ advantageous disease of DRFs as a pioneer of the medical insurance reform field [22]. Exploring the influencing factors of hospitalization cost of TCM^b^ advantageous diseases such as DRFs in TCM^a^ hospitals, can provide thoughts for the Chinese health insurance authorities to promote the reform of the payment methods for controlling medical costs in TCM^a^ hospitals, and at the same time inspire cost control of general hospitals (Western medicine) with optimization of their treatment modalities.

## Methods

### Study design and population

The study data was obtained from the Health Statistics and Information Center of the Gansu Province Health and Wellness Commission. All information on patients hospitalized in 15 TCM^a^ hospitals in Qingyang City and Tianshui City from January 2017 to June 2022 was extracted from the center’s province-wide big data platform for universal health and was cleaned and screened by the corresponding inclusion and exclusion criteria. Our inclusion criteria was western medical diagnosis code S52.500x001 (ICD-10), exclusion criteria were logical errors or missing data that could not be adjusted or supplemented based on the data, as well as patients with the length of stay greater than 90, and 1306 valid cases were included finally (Fig. S1).

### Data processing

The endogenous variables in this study were length of stay and hospitalization cost, mainly because existing studies have shown a significant correlation between hospitalization cost and length of stay [23–26], which also facilitated the subsequent comprehensive and systematic analysis of the influencing factors of hospitalization cost. The exogenous variables included patients’ basic information, medical situation, and treatment modality. Patients’ basic information included gender, ethnicity, age, marital status, complications and comorbidities, etc., indicators of medical situation included visit times, payment methods of medical insurance, hospital levels, route of admission, and treatment modalities included clinical pathways, types of treatment, use of TCM^b^ preparations, use of TCM^b^ diagnostic and therapeutic equipment, use of TCM^b^ diagnostic and therapeutic techniques, diagnosis and treatment based on TCM^b^ evidence, and surgeries and operations. In particular, since the raw data of length of stay and hospitalization cost did not obey normal distribution, the logarithm of the two data was used as the dependent variable in the regression analyses with the log-transformed data approximated a normal distribution. Further clarification, the analysis using log-transformed data aimed to explore correlations of variables theoretically, whereas the actual comparison of variances used the raw data transformed by the EXP^[log(x)]^ function. The details of the coding and assignment processing of each variable are shown in Table S1.

### Statistical analysis

Before formal statistical analysis, hospitalization cost was adjusted according to the CPI (Consumer Price Index, CPI) of Healthcare in Gansu Province from 2017 to 2022 to reduce study bias, with 2016 as the base period. Statistical analysis methods in our study mainly involve univariate analysis, multiple linear regression, and path model. Mann–Whitney U rank sum test was used in the univariate analysis when the independent variable is dichotomous, and the Kruskal–Wallis H test for a multi-categorical variable. The independent variables for multiple linear regression were selected from statistically significant variables in the univariate analysis, and regression models were built using the logarithm of length of stay and hospitalization cost as the dependent variables. It is worth mentioning that the covariate “Cities” was included in the regression analysis to minimize the bias caused by the differences in economic and social development between Qingyang City and Tianshui City. Path analysis used the logarithm of hospitalization cost as the dependent variable, the length of stay as the mediator variable, and significant independent variables from the multiple linear regression models as the input variables to comprehensively analyze the factors affecting hospitalization cost. The univariate analysis and multiple linear regression models were performed using SPSS 26.0, and the path model was developed using AMOS 24.0. The test level for the above statistical analysis was α = 0.05.

## Results

### Univariate analysis

In univariate analysis of length of stay and hospitalization cost in patients with DRFs, we found the patient’s length of stay is associated with gender, age, marital status, visit times, payment methods of medical insurance, hospital levels, admission routes, types of treatment, clinical pathways, use of TCM^a^ preparations, use of TCM^b^ diagnostic and therapeutic equipment, diagnosis and treatment based on TCM^b^ evidence, complications and comorbidities, and surgeries and operations (*P* < 0.05), and the patient’s hospitalization cost is associated with age, marital status, visit times, payment methods of medical insurance, hospital levels, admission routes, types of treatment, clinical pathways, use of TCM^a^ preparations, diagnosis and treatment based on TCM^b^ evidence, complications and comorbidities, surgeries and operations, and length of stay (*P* < 0.05) (Table 1).

**Table 1:** Description of univariate analysis of length of stay and hospitalization cost

Variables	Variable categories	N (%)	Length of stay (days)		Hospitalization cost (CNY ¥)	
			*M* (*P*_25_, *P*_75_)	*Z/H-*Value^c^ */* *P-*Value	*M* (*P*_25_, *P*_75_)	*Z/H-*Value^c^ */* *P-*Value
Gender	Male	500 (38.29%)	7.00 (4.00,10.00)	-2.548/0.011	3183.73 (1662.76, 8867.60)	-1.378/0.168
	Female	806 (61.71%)	6.00 (4.00,9.00)		2544.21 (1714.28, 8306.67)	
Nationality	Han	1270 (97.24%)	6.00 (4.00,9.00)	-1.92/0.111	2738.56 (1687.96, 8400.18)	-1.652/0.098
	Other nationality groups	36 (2.76%)	8.00 (5.00,10.75)		3429.34 (1808.06, 13043.66)	
Age(years)	< 45	470 (35.99%)	6.00 (4.00,9.00)	16.944/ < 0.001	2720.24 (1404.29, 8249.08)	11.331/0.003
	45 ~ 60	471 (36.06%)	7.00 (5.00,10.00)		2990.90 (1882.59, 8895.67)	
	> 60	365 (27.95%)	6.00 (4.00,9.00)		2503.97 (1734.64, 8098.14)	
Marital status	Unmarried	247 (18.91%)	5.00 (3.00,8.00)	26.066/ < 0.001	1964.67 (1250.60, 5529.80)	37.180/ < 0.001
	Married	888 (67.99%)	7.00 (4.00,10.00)		2890.52 (1804.28, 8394.33)	
	Others	171 (13.09%)	7.00 (5.00,10.00)		3126.52 (1664.10, 11047.07)	
Visit times	One time	1242 (95.10%)	6.00 (4.00,9.00)	-2.781/0.005	2704.91 (1671.46, 8334.98)	-3.254/0.001
	Two or more times	64 (4.90%)	8.00 (5.25,9.75)		4295.37 (2291.52, 11376.02)	
Payment methods of medical insurance	UEBMI	37 (2.83%)	9.00 (5.50,13.50)	49.485/ < 0.001	4413.34 (2186.25 10760.69)	16.511/ < 0.001
	URBMI	181 (13.86%)	7.00 (5.00,9.00)		3086.78 (1835.57, 11081.81)	
	NCMS	364 (27.87%)	7.00 (5.00,9.00)		2589.73 (1745.48, 8501.78)	
	Others	724 (55.44%)	5.00 (3.00,9.00)		2654.30(1622.24, 7632.71)	
Hospital levels	Secondary hospitals	930 (71.21%)	6.00 (4.00,8.00)	-9.192/ < 0.001	2094.13 (1535.54, 5814.56)	-12.584/ < 0.001
	Tertiary hospitals	376 (28.79%)	8.00 (5.00,13.00)		5815.86 (3076.50, 10278.23)	
Admission routes	Emergency care	145 (11.10%)	8.00 (5.00,11.00)	76.490/ < 0.001	3564.04 (2340.82, 11501.79)	91.689/ < 0.001
	Outpatient care	834 (63.86%)	6.00 (3.00,8.00)		2126.06 (1547.73, 6441.59)	
	Others	327 (25.04%)	8.00 (5.00,10.00)		4825.98 (2424.35, 9418.04)	
Types of treatment	TCM^b^ treatment	366 (28.02%)	8.00 (5.00,11.00)	55.326/ < 0.001	5309.30 (2536.97, 9705.75)	96.192/ < 0.001
	TCM^b^ and Western medical treatment	834 (63.86%)	6.00 (4.00,9.00)		2275.75 (1584.75, 6711.22)	
	Western medical treatment	106 (8.12%)	5.00 (3.00,8.25)		1960.60 (1374.33, 6865.93)	
Length of stay(days)	1 ~ 7	792 (60.64%)	4.00 (3.00,6.00)	984.631/ < 0.001	1879.37 (1407.04, 2728.60)	577.852/ < 0.001
	8 ~ 14	394 (30.17%)	9.00 (8.00,11.00)		7664.95 (3733.18, 11501.16)	
	15 ~ 21	90 (6.89%)	17.00 15.00,18.00)		11762.87 (7769.77, 16124.93)	
	22 ~ 28	20 (1.53%)	25.00 (23.00,27.75)		12064.11 (8660.13, 15612.87)	
	29 ~	10 (0.77%)	38.00 (30.50,47.75)		9477.35 (4697.18, 18214.39)	
Clinical pathways	TCM^b^ pathway	407 (31.16%)	4.00 (3.00,7.00)	154.478/ < 0.001	1919.64 (1413.24, 3440.94)	95.548/ < 0.001
	Western medicine pathway	14 (1.07%)	9.50 (6.75,15.25)		7212.22 (2610.26, 12632.69)	
	No pathway	885 (67.76%)	7.00 (5.00,10.00)		3539.02 (1876.16, 9525.20)	
Use of TCM^a^ preparations	Yes	566 (43.34%)	5.00 (3.00,8.00)	-9.998/ < 0.001	2040.52 (1503.34, 5753.35)	-8.014/ < 0.001
	No	740 (56.66%)	7.00 (5.00,10.00)		3589.26(1941.00, 9573.56)	
Use of TCM^b^ diagnostic and therapeutic equipment	Yes	1013 (77.57%)	6.00 (4.00,9.00)	-3.545/ < 0.001	2733.44 (1666.28, 8452.87)	-0.567/0.571
	No	293 (22.43%)	7.00 (5.00,9.00)		2814.87 (1796.25, 7735.17)	
Use of TCM^b^ diagnostic and treatment techniques	Yes	1073 (82.16%)	6.00 (4.00,9.00)	-1.911/0.056	2703.77 (1675.47, 8407.11)	-1.144/0.253
	No	233 (17.84%)	7.00 (5.00,9.00)		2887.65 (1792.40, 9025.56)	
Diagnosis and treatment based on TCM^b^ evidence	Yes	1164 (89.13%)	6.00 (4.00,9.00)	-2.850/0.004	2664.60 (1671.30, 8400.81)	-2.033/0.042
	No	142 (10. 87%)	7.00 (5.00,10.00)		3196.11 (1974.01, 9113.61)	
Complications and comorbidities	Yes	686 (52.53%)	7.00 (4.00,10.00)	-4.224/ < 0.001	3079.74 (1828.10, 9967.17)	-5.919/ < 0.001
	No	620 (47.47%)	6.00 (4.00,9.00)		2373.72 (1508.30, 5958.32)	
Surgeries and operations	Yes	678 (51.91%)	8.00 (6.00,11.00)	-14.352/ < 0.001	7428.57 (2558.25, 11286.71)	-19.645/ < 0.001
	No	628 (48.09%)	5.00 (3.00,7.00)		1847.35 (1385.49, 2803.50)	

*Abbreviations*:* M* (*P*_25_, *P*_75_) median (the first quartile, the third quartile), *UEBMI* Urban employee basic medical insurance, *URBMI* Urban residents’ basic medical insurance, *NCMS* New cooperative medical scheme, *TCM* Traditional Chinese medicine (TCM^a^ for ‘Traditional Chinese Medicine’, TCM^b^ for ‘diagnosis and treatment-based evidence’) ^c^Z/H-Value: Mann–Whitney U test statistical value or Kruskal–Wallis H test statistical value

### Multiple linear regression

Multiple linear regression models were established with the log-transformed values of length of stay and hospitalization cost as the dependent variables, with statistically significant in the univariate analysis as the independent variables (*P* < 0.05) (Table 2).

**Table 2:** Multiple linear regression results of length of stay and hospitalization cost of DRFs patients

Variables	Log (Length of stay)				Log (Hospitalization cost)			
	*B*^d^	Beta^e^	*t* -Value	*P* -Value	*B*^d^	Beta^e^	*t* -Value	*P* -Value
Constant	0.816		6.716	< 0.001	2.852		24.240	< 0.001
Gender (ref = Male)	-0.058	-0.096	-3.324	0.001				
Age (ref = < 45)								
45 ~ 60	0.052	0.085	2.417	0.016	-0.013	-0.016	-0.692	0.489
> 60	-0.004	-0.006	-0.181	0.856	-0.014	-0.015	-0.677	0.498
Marital status (ref = Unmarried)								
Married	0.047	0.075	2.052	0.040	0.086	0.096	3.895	< 0.001
Others	0.017	0.019	0.527	0.598	0.111	0.090	3.580	< 0.001
Visit times (ref = One time)	0.002	0.001	0.047	0.962	0.008	0.004	0.236	0.814
Payment methods of medical insurance (ref = UEBMI)								
URBMI	-0.117	-0.138	-2.371	0.018	0.040	0.033	0.825	0.409
NCMS	-0.034	-0.053	-0734	0.463	0.001	0.001	0.030	0.976
Others	-0.115	-0.196	-2.424	0.015	0.011	0.013	0.233	0.815
Hospital levels (ref = Secondary hospitals)	0.140	0.216	5.051	< 0.001	0.230	0.250	8.496	< 0.001
Admission routes (ref = Emergency care)								
Outpatient care	-0.042	-0.069	-1.610	0.108	< 0.001	< 0.001	0.009	0.993
Others	-0.021	-0.031	-0.650	0.516	0.033	0.034	1.054	0.292
Types of treatment (ref = TCM^b^ treatment)								
TCM^b^ and Western medical treatment	0.075	0.122	2.083	0.037	0.067	0.077	1.917	0.055
Western medical treatment	-0.022	-0.020	-0.469	0.639	0.042	0.028	0.933	0.351
Clinical pathways (ref = TCM^b^ pathway)								
Western medicine pathway	0.040	0.014	0.521	0.603	-0.235	-0.058	-3.154	0.002
No pathway	0.056	0.089	1.740	0.082	-0.081	-0.091	-2.608	0.009
Use of TCM^a^ preparations	-0.032	-0.054	-1.223	0.221	-0.092	-0.109	-3.678	< 0.001
Use of TCM^b^ diagnostic and therapeutic equipment	-0.036	-0.047	-1.434	0.152				
Diagnosis and treatment based on TCM^b^ evidence	0.064	0.068	2.075	0.038	0.055	0.041	2.185	0.029
Complications and comorbidities	-0.047	-0.080	-2.951	0.003	-0.045	-0.054	-2.933	0.003
Surgeries and operations	-0.176	-0.300	-10.906	< 0.001	-0.283	-0.339	-17.251	< 0.001
Cities (ref = Tianshui City)	0.049	0.084	1.934	0.053	< 0.001	-0.001	-0.018	0.986
Log (Length of stay)					0.823	0.578	30.460	< 0.001
*R*^2^ -Value	0.250				0.649			
*F* -Value	19.437				113.156			
*P* -Value	< 0.001				< 0.001			

*Abbreviations*: *UEBMI* Urban employee basic medical insurance, *URBMI* Urban residents’ basic medical insurance, *NCMS* New cooperative medical scheme, *TCM* Traditional Chinese medicine (TCM^a^ for ‘Traditional Chinese Medicine’, TCM^b^ for ‘diagnosis and treatment-based evidence’)

^d^*B* Unstandardized coefficients, ^e^*Beta* Standardized coefficients

From the results of multiple linear regression, we found the length of stay is mainly affected by the patient’s gender, age (45–60), marital status (married), payment methods of medical insurance (UEBMI, others), hospital levels, TCM^b^ and Western medical treatment, diagnosis and treatment based on TCM^b^ evidence, complications and comorbidities, and surgeries and operations, with the regression equation of the patient’s length of stay:* Y*_1_ = 0.816–0.058*X_1_ + 0.052*X_3–1_ + 0.047*X_4–1_–0.117*X_6–1_–0.115*X_6–3_ + 0.140*X_7_ + 0.075*X_9–1_–0.047*X_15_–0.176*X_16_ (*F* = 19.437, *P* < 0.001,* R*^2^ = 0.250). Hospitalization cost is mainly affected by the patient’s marital status (married, others), hospital levels, clinical pathways (Western medicine pathway, no pathway), use of TCM^a^ preparations, diagnosis and treatment based on TCM^b^ evidence, complications and comorbidities, surgeries and operations, and length of stay, with the regression equation of the patient’s hospitalization cost:* Y*_2_ = 2.852 + 0.086*X_4–1_ + 0.111*X_4–2_ + 0.230*X_7_–0.235*X_10–1_–0.081*X_10–2_–0.092 *X_11_ + 0.055*X_14_–0.045*X_15_–0.283*X_16_ + 0.823*Y_1_(*F* = 113.156, *P* < 0.001, *R*^2^ = 0.649). The *VIF* (Variance inflation factor, VIF) values for each variable in the regressions analysis of length of stay and hospitalization cost are close to or less than 10, meaning there is no collinearity in either model. Moreover, the residual statistical coefficient of hospitalization cost is $0.592\left({P}_{e}=\sqrt{{1-R}^{2}}\right)$, less than the standardized coefficient of *Y*_1_, indicating there may be other factors indirectly affecting hospitalization cost, and a comprehensive analysis of the impact of hospitalization cost should be developed by establishing a path model.

### Path model

**Fig. 1:**
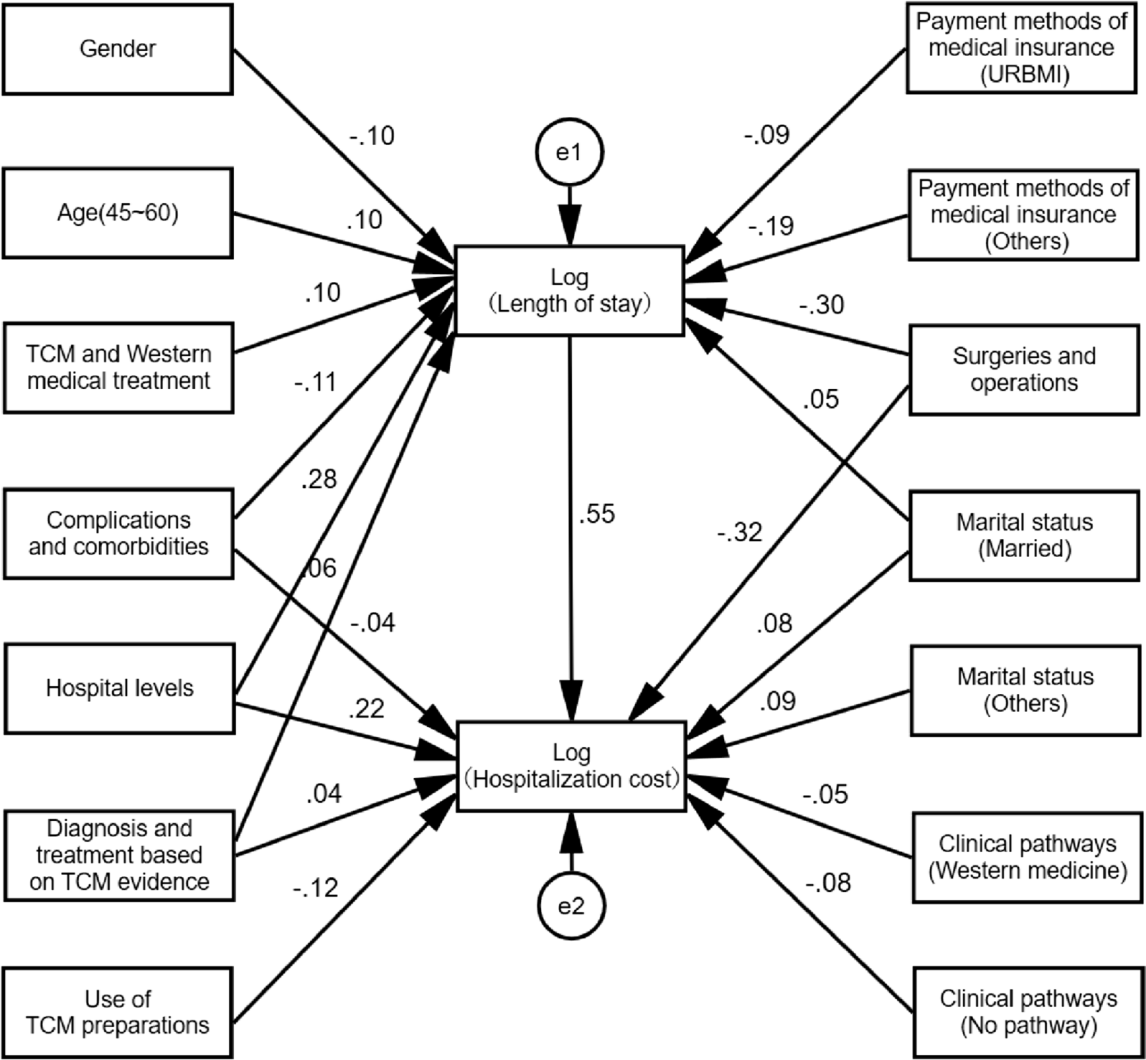
Path diagram of influencing factors of hospitalization cost of DRFs patients

Based on the multiple linear regression results of length of stay and hospitalization cost, statistically significant independent variables were included as input variables, and a path model was developed with length of stay as the mediator variable and hospitalization cost as the dependent variable (Fig. 1).

From the path model analysis results, we could get the specific decomposition effect of factors affecting the hospitalization cost of patients with DRFs, and we also could further quantitatively rank the influencing factors, the specific results are shown in Table 3. It should be stated in advance that the direct path coefficient of the independent variable on the dependent variable is equal to the standardized regression coefficient, and the indirect path coefficient of the independent variable on the dependent variable through the mediator is equal to the product of direct path coefficient of the independent variable on the mediator and direct path coefficient of the mediator on the dependent variable, and the total path coefficient is the sum of the direct path coefficient and the indirect path coefficient.

**Table 3:** Influencing factors effect decomposition table of hospitalization cost of DRFs patients

Variables	Direct effect	Indirect effect	Total effect	Ranking of total effect
Gender	—	-0.055	-0.055	12
Age (45 ~ 60)	—	0.054	0.054	13
Hospital levels	0.216	0.154	0.369	3
TCM^b^ and Western medical treatment	—	0.057	0.057	11
Complications and comorbidities	-0.042	-0.059	-0.101	7
Use of TCM^a^ preparations	-0.124	—	-0.124	4
Diagnosis and treatment based on TCM^b^ evidence	0.037	0.033	0.070	10
Payment methods of medical insurance (URBMI)	—	-0.048	-0.048	15
Payment methods of medical insurance (Others)	—	-0.107	-0.107	6
Surgeries and operations	-0.322	-0.169	-0.491	2
Marital status (Married)	0.085	0.027	0.112	5
Marital status (Others)	0.086	—	0.086	8
Clinical pathways (Western medicine)	-0.052	—	-0.052	14
Clinical pathways (No pathway)	-0.084	—	-0.084	9
Length of stay	0.554	—	0.554	1

*Abbreviations*: *URBMI* Urban residents’ basic medical insurance, *TCM* Traditional Chinese medicine (TCM^a^ for ‘Traditional Chinese Medicine’, TCM^b^ for ‘diagnosis and treatment-based evidence’)

By using the above calculation method, the effect size of the factors affecting the hospitalization cost of DRFs patients could be derived, and the ranking results of the degree of influence for each factor on the hospitalization cost as follows: length of stay, surgeries and operations, hospital levels, use of TCM^a^ preparations, marital status (married), payment methods of medical insurance (others), complications and comorbidities, marital status (others), clinical pathway (no pathway), diagnosis and treatment based on TCM^b^ evidence, TCM^b^ and Western medical treatment, gender, age (45–60), clinical pathway (Western medicine), and payment methods of medical insurance (URBMI).

## Discussion

As shown by univariate analysis, the hospitalization cost of inpatients with DRFs, an advantageous disease of Chinese medicine in TCM^a^ hospitals, was mainly related to inpatients’ age, marital status, visit times, payment methods of medical insurance, hospital levels, admission routes, types of treatment, clinical pathways, use of TCM^a^ preparations, diagnosis and treatment based on TCM^b^ evidence, complications and comorbidities, surgeries and operations, and length of stay. The hospitalization cost of patients of age (45–60) with DRFs was higher than age (< 45 or > 60), and the hospitalization cost of unmarried patients was lower than the married or other marital status. Besides, different hospitalized patients with different methods of payment for health insurance will also have an impact on their hospitalization cost, and the UEBMI was the highest, followed by the UEBMI and other health insurance, and the lowest was UEBMI, a key point to consider is that China’s township peasants’ income is lower than urban workers, and their level of health care consumption and ability are also weaker. Furthermore, the hospitalization cost through other admission routes was higher than emergency and outpatient care because patients admitted through others may have a more severe disease profile, resulting in higher consumption of medical services and resources. For example, patients admitted in the form of transfer may be transferred to higher-level hospitals because their conditions are too severe to be effectively treated in lower-level hospitals, and the medical costs the patients face in higher-level hospitals for the same diseases will be higher, as verified in our and others’ studies [27–29]. What’s more, different types of treatment for patients in TCM^a^ hospitals also led to different hospitalization cost, with the cost of pure TCM^b^ treatment being significantly higher than combined TCM^b^ and Western medicine treatment or independent Western medicine treatment, inconsistent with the results of some studies [30, 31], probably because the therapeutic effect of pure TCM^b^ treatment is relatively slow to appear, and the long treatment course leads to high cost, and the sample hospitals are TCM^a^ hospitals with mostly predominantly TCM^b^ treatment programs, making TCM^a^ cost higher. Of note, the hospitalization cost of patients without diagnosis and treatment based on TCM^b^ evidence was higher than those had, mainly because diagnosis and treatment based on TCM^b^ evidence can reduce the patient’s rehabilitation course by improving treatment efficacy and optimizing the treatment plan, resulting in relatively less hospitalization cost, consistent with the studies conducted by Shou Wujing [32], Wang Shihua [33], et al. In addition, hospitalization cost was lower for patients using TCM^a^ preparations than for those who did not, higher for patients with complications and comorbidities than for those without, and higher for patients undergoing surgery and operations than for non-surgical patients, mostly in correlation with the content of healthcare services and the consumption of healthcare resources, specifically, the use of TCM^a^ preparations speeds up the process of recovery and reduces the length of stay, and the complications and comorbidities, as well as surgeries and operations, increase the difficulty in treating the disease and generate more healthcare resources to be used.

In our study, by further combining the results of multiple linear regression and path model analysis, we found the inpatient hospitalization cost of DRFs with TCM^b^ advantageous diseases in TCM^a^ hospitals is related to length of stay, surgeries and operations, hospital levels, use of TCM^a^ preparations, payment methods of medical insurance (others), marital status (married), complications and comorbidities, marital status (other), clinical pathways (no pathway), payment methods of medical insurance (URBMI), age (45–60), clinical pathways (Western medicine), gender, and diagnosis and treatment based on TCM^b^ evidence, and the length of stay was the key influencing factor, similar to some scholars’ studies [34–37]. Simply put, the longer the length of stay, the relatively more healthcare resources are used by the hospitals, resulting in higher hospitalization cost. Additionally, hospitalization cost and length of stay were lower for female patients than for males, patients’ age (45–60) and marital status (married, other) were associated with higher hospitalization cost and length of stay, and patients would have lower hospitalization cost and length of stay if their payment methods of medical insurance are URBMI and others. Through the analysis of our models, it can also be concluded that the higher the level of the hospital, the more serious the complications and comorbidities with surgeries and operations performed, the higher hospitalization cost and the longer length of stay will be for DRFs patients, and the patients who are adopting TCM^b^ pathway, using TCM^a^ preparations, and not undergoing diagnosis and treatment based on TCM^b^ evidence would face a greater economic burden of the disease.

From the point of cost control for dominant diseases (TCM^b^ advantageous diseases) in TCM^a^ hospitals, firstly, the length of stay of patients should be minimized on the premise of ensuring the efficacy of life-saving treatment. Secondly, the rate of hospital surgery should be controlled, and the fractures that can be treated conservatively with Chinese medicine should be actively adopted [38, 39]. Thirdly, the levels of TCM^a^ hospitals, as one of the main factors influencing hospitalization cost, should receive further attention. Accordingly, the local authorities should continue to promote the construction of a hierarchical diagnosis and treatment system for TCM^a^ medical institutions, and regulate the conditions and severity of patients that should be treated in TCM^a^ hospitals of all levels reasonably, to avoid the admission of patients with lower levels of illnesses into higher-level hospitals as much as possible, and to alleviate the financial burden of illness on both the patients and the health insurance fund [40, 41]. As the main body of medical cost control, TCM^a^ hospitals should actively guide patients to use TCM^a^ preparations and carry out diagnosis and treatment based on TCM^b^ evidence, the use of TCM^a^ preparations can enable patients to get higher-value rehabilitation, and evidence-based care will enable patients to get higher-quality diagnostic and therapeutic services, both of which are conducive to the reduction of the length of stay and the realization of lower cost control. Of greater concern, the selection of clinical pathways for DRFs patients hospitalized in TCM^a^ hospitals should be based on the actual situation of the patient’s condition, and not be considered unilaterally only from the perspective of cost control, but be combined with the comprehensive consideration of patients’ treatment needs and treatment cost, with the main principle of the patients’ effective medical treatment and relatively low cost being adhered to.

## Limitations

Our study was based on hospitalized patients with DRFs in TCM^a^ hospitals in Tianshui City and Qingyang City. On the one hand, the valid samples obtained were relatively small due to the quality of the cases and other reasons, so the study was not broadly representative. On the other hand, our study mainly focused on the TCM^a^ hospitals themselves and did not incorporate the Western medicine hospitals for comparative study, making the study object too homogeneous, and it will be necessary to further optimize the content and form of the study and expand the study object and topic. In addition, the database lacks complete information on the occupations and household incomes of patients with DRFs, which may have an impact on the deeper refinement of our study.

## Conclusions

Our study indicates the main influencing factors of hospitalization cost are the length of stay, surgeries and operations, hospital levels, use of TCM^a^ preparations, payment methods of medical insurance, and complications and comorbidities, with the length of stay being the primary influencing factor. China’s government medical and health reform should pay particular attention to the length of stay of patients with TCM^b^ advantageous diseases, encourage TCM^a^ hospitals to try to take DRG or DIP as the main health insurance payment method, and advocate TCM^a^ doctors to adopt non-surgical TCM^b^ specialty therapies under the circumstance ensuring the efficacy of the treatment, to reduce the length of stay, increase health insurance reimbursement and lower the hospitalization cost as much as possible.

### Supplementary Information

**Additional file 1: Supplemental Fig. S1.** Flowchart illustrating patients selection. **Supplemental Table S1.** Classification and assignment of variables.

## Data Availability

Datasets used or analyzed during the current study are available from the corresponding author on reasonable request.

## Abbreviations

DRFs: Distal radius fractures
TCM: Traditional Chinese medicine (TCM^a^ for ‘Traditional Chinese Medicine’, TCM^b^ for ‘diagnosis and treatment-based evidence’)
DRG: Diagnosis related groups
DIP: Diagnosis-intervention packet
UEBMI: Urban employee basic medical insurance
URBMI: Urban residents’ basic medical insurance
NCMS: New cooperative medical scheme
VIF: Variance inflation factor
