# Supplementary material for: Analysis of factors influencing hospitalization cost of patients with distal radius fractures: an empirical study based on public traditional Chinese medicine hospitals in two cities, China
Source: BMC Health Serv Res. 2024 May 9;24:605. doi: 10.1186/s12913-024-10953-w (PMC11080218; doi:10.1186/s12913-024-10953-w)
Supplement: Supplementary file 1 — Additional file 1: Supplemental Fig. S1. Flowchart illustrating patients selection. Supplemental Table S1. Classification and assignment of variables. [file 12913_2024_10953_MOESM1_ESM.docx]

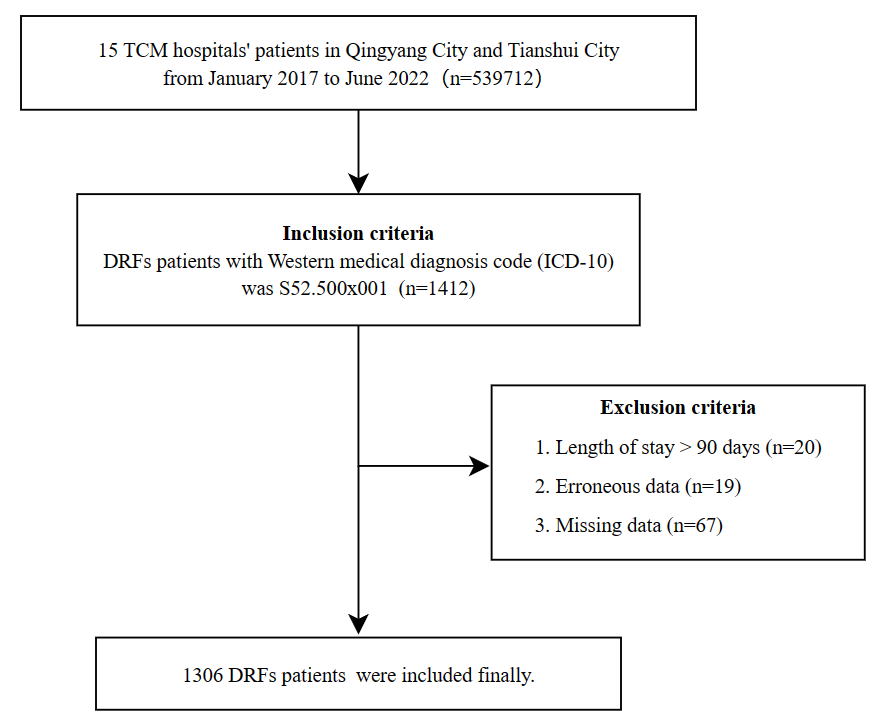


**Fig. S1** Flowchart illustrating patients selection

**Table S1** Classification and assignment of variables

| **Variable attributes** | **Variable codes** | **Variable names** | **Variable definitions** | **Dummy variables** | **Variable assignment** |
| --- | --- | --- | --- | --- | --- |
| Endogenous variables | *Y*_1_ | Length of stay (days) | The cumulative number of days from the patient's admission to discharge. | — | Log (Length of stay) |
|  | *Y*_2_ | Hospitalization cost (CNY ¥) | Medical expenses incurred when the patient is hospitalized. | — | Log (Hospitalization cost) |
| Exogenous variables | *X*_1_ | Gender | — | — | 0= Male, 1= Female |
|  | *X*_2_ | Nationality | — | — | 0=Han,  1=Other nationality groups |
|  | *X*_3-0_~*X*_3-2_ | Age(years) | — | <45 (Reference) | 0,0 |
|  |  |  |  | 45~60 | 1,0 |
|  |  |  |  | >60 | 0,1 |
|  | *X*_4-0_~*X*_4-2_ | Marital status | — | Unmarried (Reference) | 0,0 |
|  |  |  |  | Married | 1,0 |
|  |  |  |  | Others | 0,1 |
|  | *X*_5_ | Visit times | Number of hospitalizations of patients for the treatment of the same disease. | — | 0=One time,  1= Two or more times |
|  | *X*_6-0_~*X*_6-3_ | Payment methods of medical insurance | Specific ways and means of allocating health insurance cost. | UEBMI (Reference) | 0,0,0 |
|  |  |  |  | URBMI | 1,0,0 |
|  |  |  |  | NCMS | 0,1,0 |
|  |  |  |  | Others | 0,0,1 |
|  | *X*_7_ | Hospital levels | A comprehensive indicator for evaluating the qualification of hospitals based on their scales, scientific research direction, human resources and technical strength, and medical hardware and equipment. | — | 0=Secondary hospitals,  1= Tertiary hospitals |
|  | *X*_8-0_~*X*_8-2_ | Admission routes | The source of the patient's admission to the hospital for treatment. | Emergency care (Reference) | 0,0 |
|  |  |  |  | Outpatient care | 1,0 |
|  |  |  |  | Others | 0,1 |
|  | *X*_9-0_~*X*_9-2_ | Types of treatment | The medical means and methods chosen in the course of treatment. | TCM^b^ treatment (Reference) | 0,0 |
|  |  |  |  | TCM^b^ and Western medical treatment | 1,0 |
|  |  |  |  | Western medical treatment | 0,1 |
|  | *X*_10-0_~*X*_10-2_ | Clinical pathways | A set of standardized therapeutic modalities and procedures for a given disease, guided by evidence-based medical evidence and guidelines to facilitate therapeutic organization and disease management. | TCM^b^ pathway (Reference) | 0,0 |
|  |  |  |  | Western medicine pathway | 1,0 |
|  |  |  |  | No pathway | 0,1 |
|  | *X*_11_ | Use of TCM^a^ preparations | Patients use different forms of Chinese medicine products made according to Chinese medicine prescriptions. | — | 0=Yes,1=No |
|  | *X*_12_ | Use of TCM^b^ diagnostic and therapeutic equipment | Patients use TCM^b^ medical devices based on TCM^b^ diagnosis. | — | 0=Yes,1=No |
|  | *X*_13_ | Use of TCM^b^ diagnostic and treatment techniques | Patients use TCM^b^ medical techniques based on TCM^b^ diagnosis. | — | 0=Yes,1=No |
|  | *X*_14_ | Diagnosis and treatment based on TCM^b^ evidence | Through the four diagnostic methods of TCM^b^ (inspection, listening and smelling, inquiry, palpation), the Chinese medicine practitioner will analyze and identify the cause of the disease, the location of the disease, the nature of the disease, and the relationship between the evil and the positive, with a general judgment as to why the disease is present and what the evidence is, to determine the corresponding care methods. | — | 0=Yes,1=No |
|  | *X*_15_ | Complications and comorbidities | In addition to the principal diagnosis, other disorders and conditions affect medical decision-making and utilization during hospitalization. | — | 0=Yes,1=No |
|  | *X*_16_ | Surgeries and operations | Surgical and non-surgical operations (both diagnostic and therapeutic, such as interventional operations) are performed during the patient's hospitalization. | — | 0=Yes,1=No |
|  | *X*_17_ | Cities | — | Tianshui City  (Reference) | 0=Tianshui City,  1=Qingyang City |

Abbreviations: *UEBMI* Urban employee basic medical insurance, *URBMI* Urban residents' basic medical insurance, *NCMS* New cooperative medical scheme, *TCM* Traditional Chinese medicine (TCM^a^ for 'Traditional Chinese Medicine', TCM^b^ for 'diagnosis and treatment-based evidence')
